# Challenges of NTM Drug Development

**DOI:** 10.3389/fmicb.2018.01613

**Published:** 2018-07-18

**Authors:** Joseph O. Falkinham

**Affiliations:** Department of Biological Sciences, Virginia Tech, Blacksburg, VA, United States

**Keywords:** slow growth, rapid metabolism, adaptation, impermeable, hydrophobic, dormancy, biofilms, intracellular

## Abstract

Discovery and development of antibiotics active against the environmental opportunistic non-tuberculous mycobacteria (NTM) have been retarded by innate antibiotic-resistance of NTM cells and methodological challenges in the laboratory. The basis for the innate resistance of NTM cells is its lipid rich outer membrane that results in hydrophobic cells and the outer membrane’s impermeability, and the residence of NTM cells in phagocytic cells, and the slow growth and dormancy of NTM. Laboratory challenges include: the choice of species and strains for screening and measurement of anti-NTM activity, the high frequency colony switching between antibiotic-susceptible and resistant variants, the preference of NTM to adhere to surfaces and form biofilms, and the aggregation of NTM cells. Understanding these challenges can guide and inform our approaches to discovery and development of antibiotics with activity against NTM.

## Introduction to the Non-Tuberculous Mycobacteria (NTM)

The term non-tuberculous mycobacteria (NTM) encompass a large number (>150) *Mycobacterium* species that are environmental opportunistic pathogens causing pulmonary disease and skin infections in adults and cervical lymphadenitis in children (Marras and Daley, 2002). There is growing awareness of the emergence of NTM as causative agents of nosocomial infections (Marras and Daley, 2002). In the United States the species most commonly reported as causing disease are *Mycobacterium avium* subsp. *hominissuis, Mycobacterium intracellulare, Mycobacterium chimaera, Mycobacterium kansasii*, and *Mycobacterium abscessus* complex. Proven sources of infection include NTM from drinking water, potting soils, and medical equipment. Routes of infection include aerosols from water, dusts from soil, and water and soil contact with abraded or injured skin (Falkinham, 2015).

Humans are surrounded by NTM. NTM have been shown to be normal residents of drinking water distribution systems and premise plumbing; in fact, they grow in distribution systems between the treatment plant and buildings (Falkinham et al., 2001). Premise plumbing is an ideal habitat for the NTM. The water is heated and the low concentration of organic carbon is sufficient to support NTM growth. Although NTM can be grown on rich laboratory medium, they are oligotrophs able to grow on the small amount of organic matter in drinking water (George et al., 1980; Norton et al., 2004). Periods of water stagnation brought about by increased water age and oxygen consumption do not limit NTM growth, as they can grow at 12% oxygen as well as in air (21% oxygen) and can even grow, albeit at half the rate, at 6% oxygen (Lewis and Falkinham, 2015). Finally, NTM cells are surrounded by a lipid-rich outer membrane (Brennan and Nikaido, 1995) that makes NTM cells hydrophobic (van Oss et al., 1975) and impermeable (Jarlier and Nikaido, 1994). Hydrophobicity leads to the preferential attachment of NTM cells to the walls of any container or pipe, so the slowly growing NTM cells cannot be washed out. Surveys of household plumbing of *M. avium* patients showed that in 50% of the households the NTM isolates from the houses were of the same species and shared the same *rep*-PCR DNA fingerprint patterns (Falkinham, 2011). It is important to note that later work has shown that VNTR-RFLP is more discriminatory than *rep*-PCR (Iakhiaeva et al., 2016). Further, *M. avium* was common in plumbing of homes with a water heater temperatures of 125° F (50° C) or less, but rare if the water heater temperature was 130° F (55° C) or higher. That has led to a current study of the role of water heaters in increasing numbers of *M. avium* in premise plumbing. All the factors listed above undoubtedly contributed to the colonization and growth of *M. chimaera* in heater-coolers that have been shown to generate *M. chimaera*-laden aerosols and infected patients undergoing cardiac surgery (Sax et al., 2015).

## Challenges to Development of Anti-NTM Antibiotics

### Introduction

The foregoing brief introduction to the NTM is necessary before discussion of how NTM physiologic and genetic features of NTM are challenges to antibiotic development. The challenges fall into two categories: those involving difficulties in overcoming innate genetic and physiologic traits of NTM (**Table 1**) and difficulties in measuring anti-NTM antibiotic activity in the laboratory (**Table 2**). The approach that follows focuses first on laboratory challenges of susceptibility measurements and then turns to challenges due to innate NTM resistance to antibiotics. In a number of instances, it will be clear that a single NTM feature drives antibiotic resistance and laboratory measurement problems. Finally, a table is included to suggest possible solutions to the challenges (**Table 3**).

**Table 1:** Non-tuberculous mycobacteria (NTM) antibiotic development challenges for NTM due to laboratory measurement of antibiotic susceptibility.

*Mycobacterium* species strain selection
Colonial variation
Preference for surface adherence and biofilm formation
Cell aggregation
Dormancy
Residence in phagocytic cells

**Table 2:** Non-tuberculous mycobacteria antibiotic development challenges due to innate traits of NTM.

Hydrophobic cells
Lipid-rich impermeable outer membrane
Slow growth
Dormancy
Adaptation

**Table 3:** Possible solutions to challenges impeding antibiotic development for NTM.

Challenge	Possible solution
**Laboratory problem**	
Strain selection	Measure MIC in target *Mycobacterium* spp.
Colonial variation	Confirm colony type
	Initiate cultures from single colony type
Preference for adherence	Measure MIC of biofilm-grown
Aggregation	Measure MIC in early growth phase
Adaptation	Measure MIC throughout growth
Intracellular	Measure MIC in phagocytic cells
**Innate NTM trait**	
Hydrophobic cells	Amphipathic antibiotics
	Combine with OM-targeted antibiotic
Lipid-rich, impermeable OM	Amphipathic antibiotics
	Combine with OM-targeted antibiotic
Slow growth	Patience
Dormancy	Combine with resuscitation factors
Adaptation	Combine with RNA or protein synthesis inhibitor

### Laboratory Challenges

#### NTM Strain Selection

When searching among libraries of synthetic or nature compounds for anti-mycobacterial activity the choice of *Mycobacterium* spp. strains is of paramount importance. First, do not use strains of *Mycobacterium smegmatis* as indicators of potential anti-NTM activity by novel compounds in spite of their rapid rate of growth and ease of handling. Current isolates of *M. smegmatis* have been linearly cultivated for so long, that they do not resemble the original ATCC deposit. Most of the *M. smegmatis* isolates being used are derivatives of ATCC strain 607, and those lab-adapted strains have lost many unique characteristics – slow growth, hydrophobicity, and impermeability – that directly increase antibiotic susceptibility. Thus, measurements of minimal inhibitory concentrations (MIC) are not useful to guide compound selection as the *M. smegmatis* strains in use are unusually susceptible to a wide range of antibiotics. Prolonged, linear cultivation of NTM isolates selects for variants that can grow faster, have lost hydrophobicity and impermeability. Even employing *M. smegmatis* as a primary screen can be misleading, as some compounds that do not display anti-*M. smegmatis* inhibition has activity against *M. avium, M. intracellulare*, or *M. abscessus*. It is best to identify the ultimate target NTM species (e.g., *M. avium*) and measure MICs against representatives of that species (**Table 3**).

Another problem concerns selection of isolates from patients for measurement of MIC to guide and select antibiotics for therapy. More than a single isolate needs to be selected and its susceptibility measured (CSLI, 2011), as it has been shown that colonies of *M. avium* from AIDS patients having identical morphology, yielded different patterns of susceptibility to antibiotics (von Reyn et al., 1995). Further, as NTM-infected patients may be infected by more than a single NTM species as detected directly (Wallace et al., 1998) or evidenced by relapse (Boyle et al., 2016), selection of single colonies may lead to an incorrect assessment of the antibiotic of choice.

#### NTM Colonial Variation

Another problem associated with strain choice is that NTM strains can display alternative colonial variants. For *M. avium* and *M. intracellulare*, isolates exhibit either a transparent or opaque colony form (McCarthy, 1970; Stormer and Falkinham, 1989). Likewise, *M. abscessus* complex isolates can exhibit either a rough and smooth colonial morphology (Howard et al., 2006). In *M. avium* and *M. intracellulare* the transparent (T) “Mexican Hat” colony morphology is found upon primary culture from patients. The transparent (T) variant is slow-growing, hydrophobic, virulent, and antibiotic-resistant compared to the opaque (O) variant that emerges during laboratory medium cultivation (Schaefer et al., 1970). The opaque (O) variants are faster growing, less hydrophobic, and antibiotic-sensitive compared to the isogenic transparent (T) variants. In *M. avium*, single colony isolates switch between the T and O forms; colony variation is fully reversible (McCarthy, 1970). The frequency of colony variants of the opposite type amongst isolates of *M. avium* is approximately 1 in 1,000 (Stormer and Falkinham, 1989). Thus, in a barely turbid culture of one-hundred million (10^8^) *M. avium* (T) variant cells, there are approximately one-hundred thousand (10^5^ O) cells. Thus, special care must be taken to sample every culture to ensure that the frequency of colonies of the opaque (O) type are always less than or equal to 1 in 1,000 (**Table 3**).

As the alternative colonial variants have different susceptibilities to antibiotics (McCarthy, 1970; Stormer and Falkinham, 1989), measurement of MIC values may not provide the guidance to direct either chemotherapy or selection of promising novel anti-mycobacterial compounds. For example, as transparent (T) colony variants predominate in patients, selection of an isogenic opaque (O) variant for MIC measurement will lead to choice of an antibiotic that may lack activity against the patient’s transparent (T) variant. This may be the basis, in part, for the lack of any correlation between MIC measurements and prediction of therapeutic success (Sison et al., 1996; Van Engen et al., 2010; Schön and Chryssanthou, 2017). It might also result in the advancement of a compound for further testing based on MIC values from an opaque (O) variant, that will be ineffective in patients as the transparent variant (T) is resistant to the compound.

#### NTM Hydrophobicity and Measurement of Antibiotic Susceptibility

Non-tuberculous mycobacteria hydrophobicity also creates problems in measuring MICs to guide compound selection or patient therapy. As NTM are hydrophobic, they prefer surface attachment and growth rather than replicating in aqueous suspension. That means that the NTM cells are not in suspension, but rather adhere to the walls of the individual wells in 96-well plates used to measure MICs as recommended by Clinical and Laboratory Standards Institute (CSLI, 2011). Measurements of NTM cells adhering to the walls of water heaters and pipes in households and hospitals, in medical equipment (e.g., heater-coolers), and in test tubes in the laboratory shows that the majority of NTM cells (>99%) are surface-attached (Falkinham et al., 2001; Falkinham, 2011). This observation alone provides a reason why measurement of antibiotic susceptibility in tubes or 96-well plates has not provided guidance for therapeutic efficacy in patients (Sison et al., 1996; Van Engen et al., 2010; Schön and Chryssanthou, 2017). Simply, measurement of turbidity of cell suspensions does not accurately reflect the number of total cells; most are not in suspension but adhering to the wells or tubes. The solution is to use methods to measure MIC of biofilm-grown cells in biofilms (**Table 3**).

Non-tuberculous mycobacteria preference for adherence and biofilm-formation (Falkinham et al., 2001; Falkinham, 2011) also explain why disinfection of hospital pipes and medical equipment generally fails. The NTM cells in the biofilms survive disinfection and can re-inoculate the water.

A second hydrophobicity-driven problem reducing the utility of standard methods of MIC measurement (CSLI, 2011) is the spontaneous aggregation of NTM cells. Typically, early growth of NTM cells in laboratory medium (e.g., M7H9 broth) can be followed by increases in turbidity, but upon reaching the mid to late exponential phase of growth turbidity disappears and visible aggregates of various dimensions appear. Thus, for many NTM isolates, antibiotic susceptibility cannot be measured with accuracy. Aggregation between NTM cells is driven by hydrophobicity, just as hydrophobicity drives the adherence of NTM to the walls of tubes and wells (van Oss et al., 1975). The best solution to this problem is to measure MIC of isolates during the early phases of cell growth, before aggregation occurs (**Table 3**). This may require amplification, such as PCR or dyes, to increase the signal from the low cell numbers.

There are no other current useful approaches to reduce the aggregation of NTM cells, as the use of detergent reduces cell-surface hydrophobicity and abolishes aggregation, but increases NTM permeability and growth rate, and thereby the susceptibility to antibiotics. Selection of non-aggregating variants of NTM isolates is not a solution, as the non-aggregating variants have reduced cell hydrophobicity and altered outer membrane composition. Thus, the cells are not representative of those recovered from the patient. This is represented by the laboratory-culture-induced selection of opaque (O) variants of the transparent (T) variants that are isolated from patients.

#### NTM Adherence and Biofilm Formation

Non-tuberculous mycobacteria cells prefer surface adherence to residence in suspension, whether in humans, in water, or in laboratory growth medium. Surface attachment allows NTM cells to reduce the interaction of their hydrophobic surfaces with the positive and negative charges in aqueous suspension. Once the NTM cells adhere, they can grow to form a biofilm that consists of NTM and other microbial cells within a polymeric matrix consisting of polysaccharide, lipid, DNA, and protein (Mullis and Falkinham, 2013). The layers of cells within the matrix are relatively impermeable to antibiotics and disinfectants. There are significant differences in the susceptibility to chlorine (Falkinham, 2003; Steed and Falkinham, 2006) or antibiotics (Falkinham, 2007) of *M. avium* cells grown and exposed in biofilms compared to suspension-grown and exposed cells. The increased resistance of *M. avium* cells in biofilms to antimicrobial agents, compared to those grown and exposed in suspension, prevents obtaining an accurate measurement of disinfectant- or antibiotic-susceptibility for guidance in water treatment or patient care. Again, the solution to biofilm formation is to measure MICs of NTM cells grown in biofilms (**Table 3**).

That is not the only problem of NTM biofilm formation. Surprisingly, cells of *M. avium* grown in biofilms, yet harvested and exposed as single cell suspensions were significantly more tolerant of chlorine or antibiotics than cells grown and exposed in suspension (Steed and Falkinham, 2006; Falkinham, 2007). That tolerance was transient, as cells lost the increased resistance to chlorine or antibiotics exhibited by suspension-grown cells following overnight growth in fresh medium (Steed and Falkinham, 2006; Falkinham, 2007). I speculate that such a physiologic adaptation (adaptation, because cells are not permanently altered in susceptibility) is a consequence of growth of NTM-cells in a biofilm.

#### NTM Dormancy

NTM-dormancy has been demonstrated in a variety of NTM species, particularly in *M. avium* (Archuleta et al., 2005). *M. avium* cells subjected to nutrient starvation entered a dormant stage in which cells are effectively non-growing and thereby resistant to any antibiotic challenge (Archuleta et al., 2005). Whether such NTM-dormant cells are in patient tissue or in the wells of an antibiotic-containing medium, such non-growing cells would be expected to be resistant to most antibiotics, as the antibiotics do not actively destroy cell components.

As demonstrated by the studies of Larry Wayne, the slow decrease in oxygen levels as *M. tuberculosis* triggers the formation of lung tubercles, points out that dormancy of *M. tuberculosis* is triggered by the absence of oxygen. Those dormant cells can, under certain conditions, be triggered to grow again. The discovery of proteins that can induce growth in such dormant cells, suggests an approach to measurement of MIC of cells suspected to be dormant; namely resuscitate the dormant cells (Wivagg and Hung, 2012) and measure the MIC following the subsequent growth (**Table 3**).

#### NTM Residence in Phagocytic Cells

Non-tuberculous mycobacteria cells are not only found within phagocytic cells in infected individuals, but also in granulomas in infected organs like the lungs and liver. NTM-cells in granulomas have not only the NTM outer membrane to protect them, but they also have the layers of host, human cells that make up the granuloma. When considering that it is likely that the majority of NTM-cells are located within mammalian phagocytic cells in infected individuals, a problem of permeability re-appears. Here, it is not the NTM cell’s impermeability that is the only factor, but rather the impermeability of the phagocytic cell’s membrane now plays a role. Recognition of that fact led David Yajko and his colleagues to measure antibiotic susceptibility of NTM cells in human macrophages (Yajko et al., 1991). However, as discussed above, impermeability can be possibly overcome using combinations of agents; namely, one agent to increase the permeability of the phagocytic cell’s membrane to achieve bacteriostatic or bactericidal concentrations within those cells and a second for killing or inhibiting the growth of the NTM cells (**Table 3**). Documentation that the MICs of the intracellular NTM cells were higher than those of the NTM cells alone, forces us to consider this approach.

### Innate Resistance of NTM to Antibiotics

#### NTM Hydrophobicity and Impermeability and NTM Susceptibility to Antibiotics

Both the hydrophobic and impermeable NTM outer membrane are major and independent contributors to the lack of susceptibility of NTM to commonly used antibiotics (Rastogi et al., 1981; Jarlier and Nikaido, 1994; Brennan and Nikaido, 1995). NTM are the most hydrophobic of bacteria (van Oss et al., 1975), due to the fact that cells are surrounded by the lipid-rich outer membrane that comprises 30% of the cell mass (Brennan and Nikaido, 1995). Hydrophobicity, measured as water droplet contacts angles on filters covered with NTM cells are high (≥75°) almost resembling water droplets on a freshly waxed automobile. The non-polar cell surface prevents the adherence or binding of antibiotics that carry positive or negative charges.

The lipid-rich outer membrane is also impermeable. Measurement of rates of uptake of compounds showed the uptake of charged antibiotics, nutrients, metals, oxyanions, and disinfectants was only 1% of the rate measured in rapidly growing bacteria such as *Escherichia coli* (Jarlier and Nikaido, 1994). Thus, not only is adherence of charged compounds reduced, but the rate of transport though the outer membrane is greatly reduced. Therefore, it is not surprising that NTM exhibit resistance to most commonly used antibiotics. That having been stated, it is important to point out that the targets for antibiotic action, for example the 23S rRNA for the erythromycin-family antibiotic clarithromycin, are intact and resemble those in other bacteria. That can be shown by exposing NTM cells to antibiotics or other anti-microbial compounds in the presence of detergent. Detergent disrupts the outer membrane, reducing hydrophobicity and increasing permeation, artificially creating phenocopy cells that are antibiotic-susceptible.

One successful route to development of anti-NTM antibiotics is to synthesize hydrophobic derivatives of existing antibiotics. That approach was taught in a paper describing the synthesis of effective anti-NTM drugs through the addition of hydrophobic groups, starting with an ineffective and hydrophilic drug (Rastogi et al., 1988). Although those drugs are not in use, there is the example of synthesizing hydrophobic derivatives of erythromycin, a common antibiotic with little activity against NTM. Hydrophobic derivatives of erythromycin, namely clarithromycin and azithromycin have been shown effective in treating NTM-infected patients whether suffering from NTN pulmonary disease or NTM bacteremia. Following that rational for anti-NTM antibiotic development, a family of Amphipathic dendritic amphiphiles were synthesized and their anti-NTM activity measured (**Table 3**). These compounds consist of a fatty acid tail linked to (Williams et al., 2007; Falkinham et al., 2012). A number of these compounds displayed strong antibiotic activity against *M. avium, M. intracellulare*, and *M. abscessus* with MICs all below 10 μg/mL (Williams et al., 2007; Falkinham et al., 2012).

Another successful approach to overcoming the hydrophobic barrier of the NTM outer membrane is through combinations of antibiotics (**Table 3**). For example, the antibiotic ethambutol is an inhibitor of formation of the arabinogalactan polymer that links the NTM outer membrane to the peptidoglycan cell wall. Although ethambutol is not a first line drug, it acts synergistically with other antibiotics with targets within cells. Ethambutol reduces the hydrophobic and impermeable outer membrane barrier resulting in increased transport of the second drug (Yajko et al., 1988). That demonstrated example of rational synergism, suggests that the search for anti-NTM antibiotics includes screening methods to identify those that target and disrupt the outer membrane and then measure MICs of those compounds in combination with drugs whose targets are intracellular. Adoption of this view strongly suggests that screening natural and synthetic compounds for anti-NTM activity include all possible combinations and screening in combination with known, albeit low, anti-NTM active antibiotics such as ethambutol.

#### NTM Slow Growth and Adaptation

The unique structural feature of the mycobacteria; namely the presence of a long chain lipid and wax-rich outer membrane, is the major determinant of their physiology, growth, ecology, and epidemiology (Brennan and Nikaido, 1995). As the outer membrane makes up 30% of the total mycobacterial cell weight, its synthesis reduces energy available for production of new cells. Consequently, NTM grow slowly with a generation time of 1 doubling per day under nutrient-rich conditions at 37°C. However, it is important to point out that the NTM do not replicate slowly because of a slow rate of metabolism. NTM metabolism, as reflected by oxygen uptake and ATP production, is as active as that of rapidly growing bacteria; the NTM simply expend a great deal of energy making the outer membrane. That rapid rate of metabolism means that NTM cells can induce gene products that protect against environmental stress before cells divide.

In *M. avium*, growth rate modulates antibiotic susceptibility. The susceptibility of *M. avium* cells to antibiotics or chlorine is greater in medium that supports more rapid growth compared to a minimal, nutrient-poor medium (Falkinham, 2003). In measuring antibiotic MICs of NTM isolates, it is valuable to examine and identify MICs on a daily basis (**Table 3**). For example, inspection of MICs for the antibiotic clarithromycin against strains of *Mycobacterium abscessus* identified some whose MIC values increased over time. Significantly, the MIC measured after 3 days incubation was significantly lower than that measured at 7 days (Nash et al., 2009). Further examination of that behavior led to the discovery of an erythromycin methylase that protected cells from inhibition of protein synthesis due to its induction and synthesis leading to methylation of the 23S rRNA target of clarithromycin (Nash et al., 2009).

The studies of Maaløe and Kjeldgaard (1966) taught microbiologists the concept of “balanced” and “unbalanced” growth and the relationship of growth rate to the efficacy of antibiotics. Growth of microorganisms in the absence of antibiotics leads to “balanced” conditions where the increases in the amount of DNA, RNA, protein, and cell walls are proportionate with the growth rate. Exposure of cells growing under “balanced” conditions to an antibiotic unbalances growth. For example, as antibiotics have a single target (e.g., DNA polymerase), it follows that exposure to an antibiotic that inhibits the activity of DNA polymerase would lead to an “unbalanced” condition where increases in of DNA, are not proportionate with increases in cell mass. “Unbalanced” growth of microbial cells leads to death, particularly in rapidly dividing cells (e.g., *E. coli*). However, unlike *E. coli*, NTM cells can react to antibiotic stress by synthesizing proteins and other cell constituents able to protect the cells before they are forced to divide (24 h/generation).

To explain NTM-adaptation further, what follows are the results of a study of *M. avium* heat-adaptation. My students and I have found heat-tolerance in *M. avium* isolates recovered from plumbing biofilm samples collected from homes of *M. avium*-infected patients. The isolates are able to survive exposure to 60°C for 3 h without any loss in colony-forming units; they are only killed at 65°C. This data is in contrast to published data on heat-susceptibility of NTM species (Schulze-Röbbecke and Buchholtz, 1992). As the patient, plumbing, and water-provider *M. avium* isolates shared an identical VNTR-RFLP DNA fingerprint patterns (Iakhiaeva et al., 2016) and heat-tolerance was an adaptation, we hypothesized that passage through the home’s water heater may have triggered an adaptive response leading to increased heat-tolerance. Accordingly, we grew the household *M. avium* strains at 25°, 30°, 35°, and 42° (the latter the highest temperature of growth for *M. avium*) and measured their susceptibilities to 55°, 60°, and 65°C. Further, we measured the cell concentration of trehalose that has been shown to be a modulator of temperature-susceptibility. Compared to cells grown at 25°C, cells grown at 42°C had 10-times more trehalose/cell protein. Further, cells grown at 42°C were significantly more tolerant to survival at 60°C compared to those grown at 25°C.

## Concluding Comments

The foregoing information provides strong support for the notion that the search for novel anti-NTM antibiotics will continue to be difficult. It is already well-established that laboratory measures of antibiotic MICs of suspension-grown NTM cells do not provide guidance for therapy (Sison et al., 1996; Van Engen et al., 2010; Schön and Chryssanthou, 2017). The challenges involve both methods of measuring antibiotic susceptibility of NTM strains in the laboratory (**Table 1**) as well as developing novel anti-NTM antibiotics that can overcome innate characters of these waterborne opportunistic pathogens (**Table 2**). I have suggested several solutions to these problems (**Table 3**), but further approaches are needed.

First, identification of suitable novel anti-NTM antibiotics requires development of accurate laboratory measures of NTM antibiotic susceptibility. That requires choice of species and strains coupled with the development of accurate measures of suspension-, biofilm-, and intracellular- (e.g., macrophage) NTM susceptibility (**Table 1**). Those *in vitro* measures must be supplemented with ways to prevent NTM cell aggregation, without altering NTM susceptibility.

The second challenge facing development of anti-NTM antibiotics involves efforts to overcome NTM cell surface hydrophobicity and outer membrane impermeability (**Table 2**). A starting point might be the realization that successful anti-NTM drug therapy involves synergistic combinations; one antibiotic to disrupt or inhibit the outer membrane, and a second antibiotic to inhibit a critical cellular process (i.e., DNA, RNA, or protein and outer membrane synthesis) and hence unbalance growth. For such an approach, high throughput screening involving pairs or compounds seems the likely tool.

## Author Contributions

JF researched, practiced, and wrote the article.

## Conflict of Interest Statement

The authors declare that the research was conducted in the absence of any commercial or financial relationships that could be construed as a potential conflict of interest.
